# Enzymatic Synthesis of Magnetic Nanoparticles

**DOI:** 10.3390/ijms16047535

**Published:** 2015-04-03

**Authors:** Arati G. Kolhatkar, Chamath Dannongoda, Katerina Kourentzi, Andrew C. Jamison, Ivan Nekrashevich, Archana Kar, Eliedonna Cacao, Ulrich Strych, Irene Rusakova, Karen S. Martirosyan, Dmitri Litvinov, T. Randall Lee, Richard C. Willson

**Affiliations:** 1 Department of Chemistry and Texas Center for Superconductivity, University of Houston, Houston, TX 77204, USA; E-Mails: akolhatkar@uh.edu (A.G.K.); ajamison@uh.edu (A.C.J.); 2 Department of Physics and Astronomy, University of Texas at Brownsville, Brownsville, TX 78520, USA; E-Mails: chamath.dannangoda@gmail.com (C.D.); Karen.Martirosyan@utb.edu (K.S.M.); 3 Department of Chemical and Biomolecular Engineering, University of Houston, Houston, TX 77204, USA; E-Mails: edkourentzi@uh.edu (K.K.); archana.kar@gmail.com (A.K.); eecacao@gmail.com (E.C.); 4 Department of Electrical and Computer Engineering, University of Houston, Houston, TX 77204, USA; E-Mails: inekrashevich@gmail.com (I.N.); litvinov@uh.edu (D.L.); 5 Department of Biology and Biochemistry, University of Houston, Houston, TX 77204, USA; E-Mail: ustrych@gmail.com; 6 Department of Physics and Texas Center for Superconductivity, University of Houston, Houston, TX 77204, USA; E-Mail: rusakova@uh.edu; 7 Centro de Biotecnología FEMSA, Departamento de Biotecnología e Ingeniería de Alimentos, Tecnológico de Monterrey, Campus Monterrey, Monterrey, NL 64849, Mexico

**Keywords:** enzymatic synthesis, magnetic nanoparticles, alkaline phosphatase, magnetic sensing

## Abstract

We report the first *in vitro* enzymatic synthesis of paramagnetic and antiferromagnetic nanoparticles toward magnetic ELISA reporting. With our procedure, alkaline phosphatase catalyzes the dephosphorylation of l-ascorbic-2-phosphate, which then serves as a reducing agent for salts of iron, gadolinium, and holmium, forming magnetic precipitates of Fe_45±14_Gd_5±2_O_50±15_ and Fe_42±4_Ho_6±4_O_52±5_. The nanoparticles were found to be paramagnetic at 300 K and antiferromagnetic under 25 K. Although weakly magnetic at 300 K, the room-temperature magnetization of the nanoparticles found here is considerably greater than that of analogous chemically-synthesized Ln*_x_*Fe*_y_*O*_z_* (Ln = Gd, Ho) samples reported previously. At 5 K, the nanoparticles showed a significantly higher saturation magnetization of 45 and 30 emu/g for Fe_45±14_Gd_5±2_O_50±15_ and Fe_42±4_Ho_6±4_O_52±5_, respectively. Our approach of enzymatically synthesizing magnetic labels reduces the cost and avoids diffusional mass-transfer limitations associated with pre-synthesized magnetic reporter particles, while retaining the advantages of magnetic sensing.

## 1. Introduction

Interest in magnetic biosensing has grown tremendously over the past decade. Magnetic nanoparticles (MNPs), commonly used in sample capture, clean-up, and concentration, are also now evaluated as labels for sensitive biomolecule detection [1] since they are unaffected by photobleaching or turbidity, and magnetic background is ubiquitously absent even from the most complex biological samples. The application of giant magnetoresistive (GMR) sensors and MNP labels to bioassays and diagnostics was first suggested by Baselt *et al.* in 1998 [2], and by Shieh and Ackley in 2000 [3]. This approach is attractive because of the solid-state and potentially low-cost nature of the sensors, and the absence of concerns associated with photobleaching, scattering, and fouling. Research groups at the University of Minnesota [4,5,6] and at Stanford University [7,8,9,10] have reported micrometer-scale magnetic sensors for ultrasensitive protein detection in complex samples. Moreover, several magnetic immunoassays integrated with proprietary readers have been commercialized, including those from MagArray [11], MagniSense [12], and MagnaBiosciences [13].

**Figure 1:**
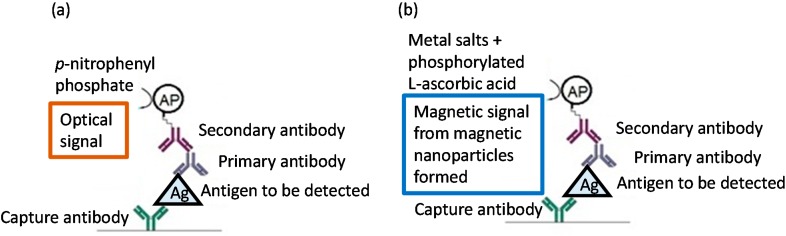
(**a**) Conventional ELISA—detection by optical signal (**b**) our novel strategy—detection by magnetic signal. Figure adapted from [23].

Conventional enzyme-linked immunosorbent assays (ELISAs) rely on modification of a substrate to form a detectable product that absorbs light, fluoresces, or luminesces. For example, *p*-nitrophenyl phosphate is dephosphorylated by alkaline phosphatase (AP) to form a soluble yellow product (*p*-nitrophenol) that is readily detected at 405 nm using a spectrophotometer (Figure 1). The substrates 4-methylumbelliferyl phosphate (4-MUP) and 3-(2'-spiroadamantane)-4-methyl-4-(3'-phosphoryloxyphenyl-1,2-dioxetane, disodium salt (AMPPD) are likewise dephosphorylated by AP to fluorescent and luminescent products, respectively. Where an insoluble colored product is necessary, AP dephosphorylation of bromochloroindoyl phosphate-nitroblue tetrazolium (BCIP-NBT) leads to the formation of a blue precipitate/chromophore [14,15]. Enzymes also can produce silver, as in silver staining, using the redox chemistry underlying black and white photography. The recent application of such staining technology includes the enzyme-mediated formation of silver nanoparticles [16,17,18,19]. AP can produce metallic silver by dephosphorylation of an appropriate substrate (e.g., l-ascorbic-2-phosphate [19], *p*-aminophenyl phosphate [20], and 3-indoxyl phosphate [21]) that acts as a reducing agent.

In this paper, we report the first enzymatic synthesis of insoluble magnetic material for use in biosensing and also in materials science. In this approach, AP catalyzes the dephosphorylation of l-ascorbic-2-phosphate, which then reduces iron, gadolinium, and holmium chlorides to yield paramagnetic MNPs at room temperature. Our strategy offers a novel approach to magnetic sensing in which the magnetic reporter can be enzymatically synthesized *in situ*. In contrast to conventional sandwich ELISA that uses an optical read-out, in our method (Figure 1), the substrate is dephosphorylated by alkaline phosphatase to yield a magnetic product that can be detected using a giant magnetoresistive (GMR) sensor with a much higher sensitivity [5,6,7,22]. This approach is inexpensive and circumvents the substantial mass-transfer concerns associated with pre-synthesized magnetic reporter particles while preserving the advantages of magnetic sensing, including the use of inexpensive solid-state detectors and the elimination of optical sensing challenges. This work also represents the first demonstration of the enzymatic synthesis of (albeit weakly) magnetic nanoparticles.

## 2. Results and Discussion

In nature, magnetotactic bacteria [24] possessing specialized organelles (magnetosomes) have the ability to synthesize ferrimagnetic crystals of either magnetite (Fe_3_O_4_) or the iron sulfide greigite (Fe_3_S_4_). The synthesis of these magnetic particles is encoded by at least 28 different genes, [25] and translating this natural synthesis approach to the bench with high yields and magnetization has been challenging [26]. In contrast, our approach uses a single enzyme to form magnetic material. In initial efforts to obtain Fe–Gd–O and Fe–Ho–O precipitates through enzymatic means, we explored the chemical reduction of various metal salts. Although the metal salts were reduced, the precipitates formed were non-magnetic (details in the Experimental Section). Furthermore, although gadolinium and holmium are common components of permanent magnets, reduction to these rare-earth elements from their chloride salts alone failed to yield magnetic precipitates.

The introduction of dopants during the chemical synthesis of MNPs has been previously demonstrated [27,28,29]. Johnson *et al.* found that ZnO nanoparticles lacking a doping metal exhibit weak or no magnetic properties, but when Fe was used as a dopant, the resulting Zn_1−*x*_Fe*_x_*O product showed noticeable levels of magnetization that increased as Fe was increased from 0% to 10% [28]. In our screening experiments, we observed that 6:1 molar ratio mixtures of ferric chloride and either gadolinium chloride or holmium chloride gave precipitates that were attracted to a bar magnet. We then enzymatically converted l-ascorbic-2-phosphate to l-ascorbic acid and found that the latter could serve as a reducing agent for iron, gadolinium, and holmium salts. The resulting precipitates were magnetic. In our novel enzymatic process, gadolinium and holmium are incorporated into the products as dopants, producing measurable magnetic properties as compared to the non-magnetic iron oxide precipitate formed in the absence of these dopants. The synthesis conditions and characterization methods are described in detail in the Experimental Section.

**Figure 2:**
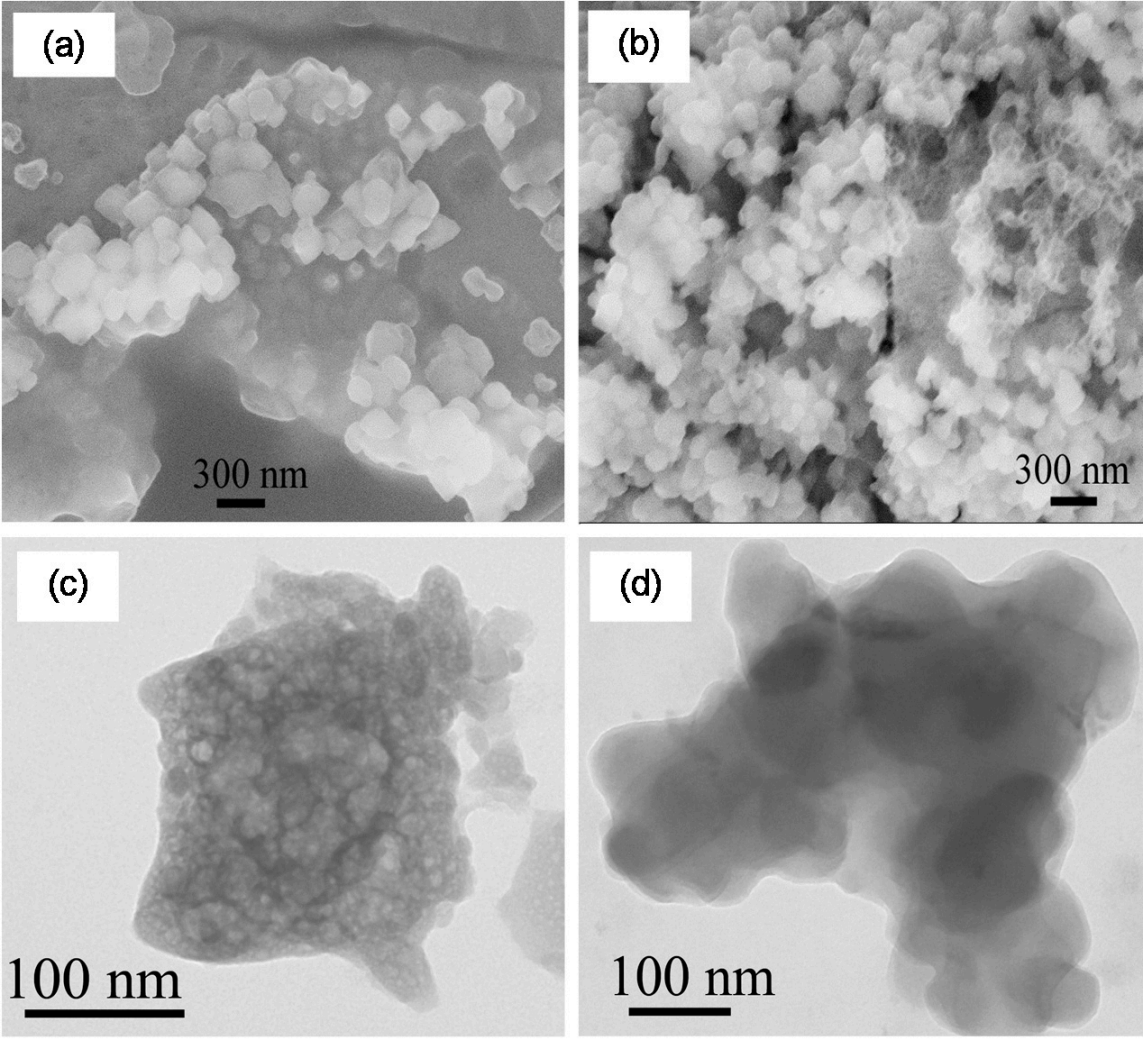
Microscopy images of chemically-synthesized magnetic nanoparticles: SEM (**a**) Fe–Gd–O; (**b**) Fe–Ho–O; and TEM (**c**) Fe–Gd–O; (**d**) Fe–Ho–O.

**Figure 3:**
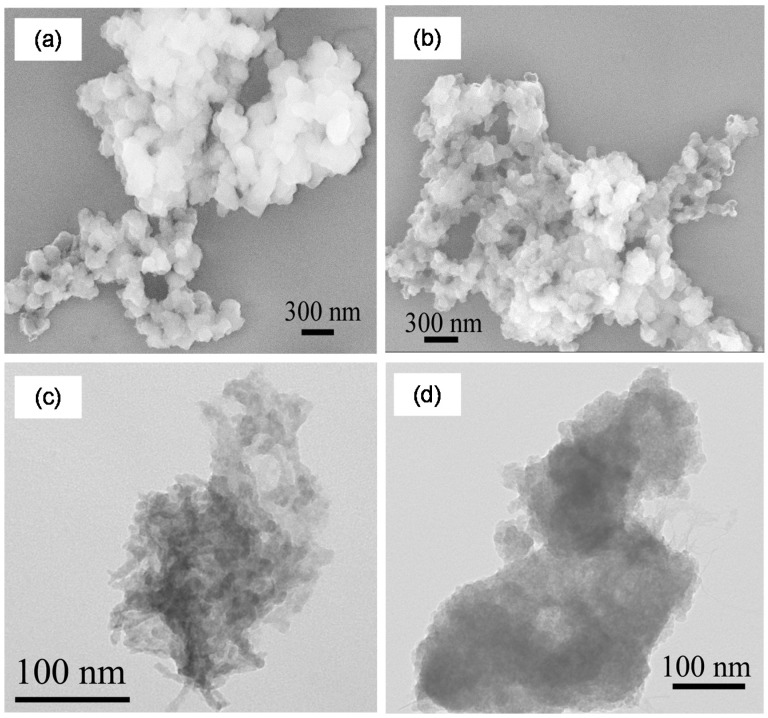
Microscopy images of enzymatically-synthesized magnetic nanoparticles: SEM (**a**) Fe–Gd–O; (**b**) Fe–Ho–O; and TEM (**c**) Fe–Gd–O; (**d**) Fe–Ho–O.

Scanning electron microscopy (SEM) and transmission electron microscopy (TEM) images of the chemically- and enzymatically-synthesized MNPs are shown in Figure 2 and Figure 3, respectively. The sizes of the MNPs are in the range of 100–150 nm. Elemental composition was determined using SEM/energy dispersive X-ray (EDX) spectroscopy and TEM/EDX as described in the Experimental Section. The chemically-synthesized nanoparticles were found to be Fe_43±18_Gd_2±0_O_55±18_ and Fe_3±1_Ho_11±2_O_85±3_, while the enzymatically-synthesized nanoparticles were composed of Fe_45±14_Gd_5±2_O_50±15_ and Fe_42±4_Ho_6±4_O_52±5_.

Elemental mass balances on the synthetic process were estimated from the following data. An aliquot (80 μg; 0.9 units) of AP protein gave magnetic precipitates of 80 mg Fe_45±14_Gd_5±2_O_50±15_ and 90 mg Fe_42±4_Ho_6±4_O_52±5_ for each of the MNP syntheses (*i.e.*, 1 µg protein used for 1 mg NP synthesized). Based on the EDX data, the weight % ratio of Fe:Gd and Fe:Ho was (59 ± 14):(20 ± 2) and (57 ± 9):(23 ± 12), respectively. The initial masses of Fe, Gd, Ho were 40 mg, 20 mg, and 20 mg, respectively. Taking a mass balance with respect to iron, gadolinium, and holmium, recovery was estimated at 118% ± 28% and 80% ± 8% for Fe and Gd in the Fe–Gd–O precipitate and 128% ± 20% and 100% ± 54% for Fe and Ho in Fe–Ho–O, where the non-homogeneous composition likely led to significant deviations in the measured recovery.

**Figure 4:**
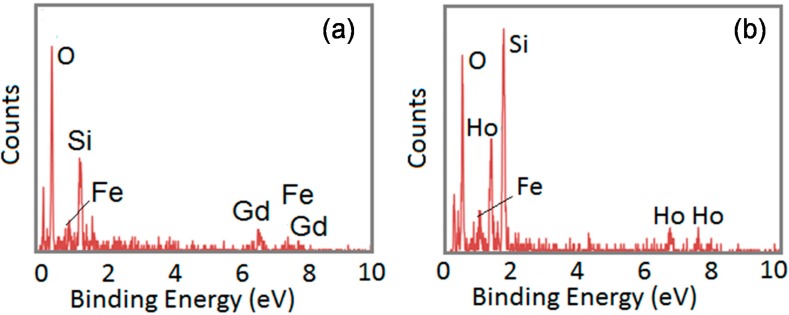
SEM/EDX analysis of the chemically-synthesized (**a**) Fe_43__±18_Gd_2__±0_O_55__±18_ and (**b**) Fe_3__±1_Ho_11__±2_O_85__±3_ magnetic nanoparticles.

**Figure 5:**
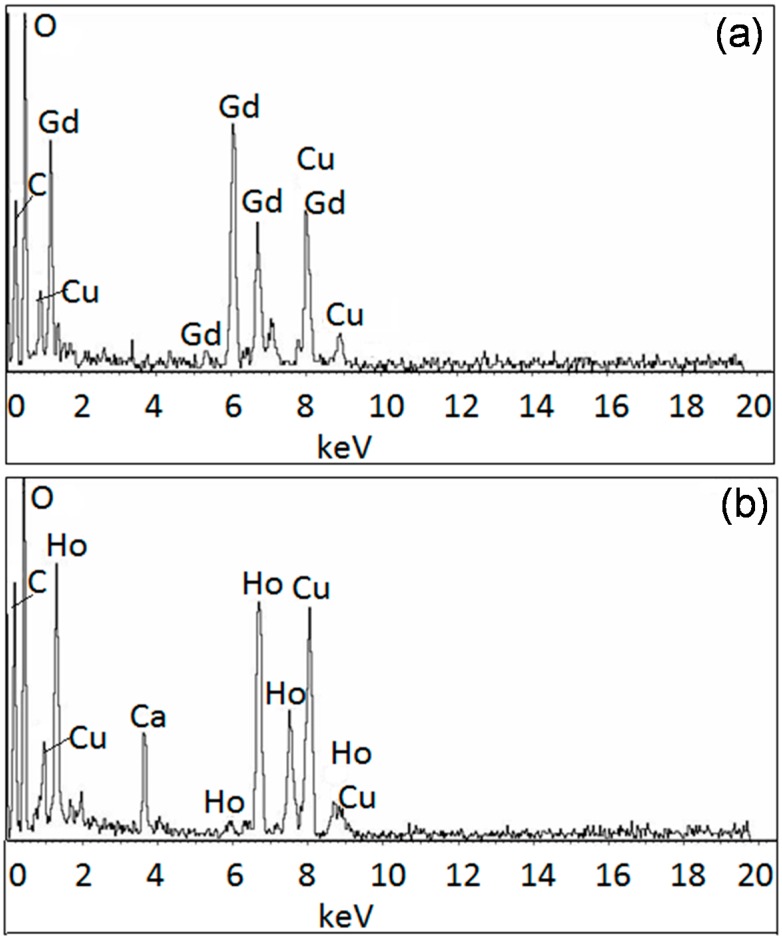
TEM/EDX analysis of the chemically-synthesized (**a**) Fe–Gd–O and (**b**) Fe–Ho–O magnetic nanoparticles.

**Figure 6:**
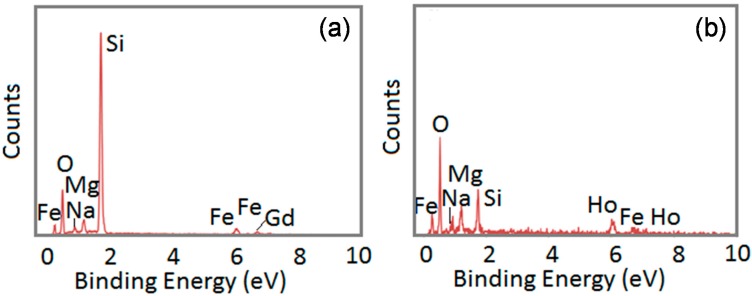
SEM/EDX analysis of the enzymatically-synthesized (**a**) Fe_45__±14_Gd_5__±2_O_50__±15_ and (**b**) Fe_42__±4_Ho_6__±4_O_52__±5_ magnetic nanoparticles.

**Figure 7:**
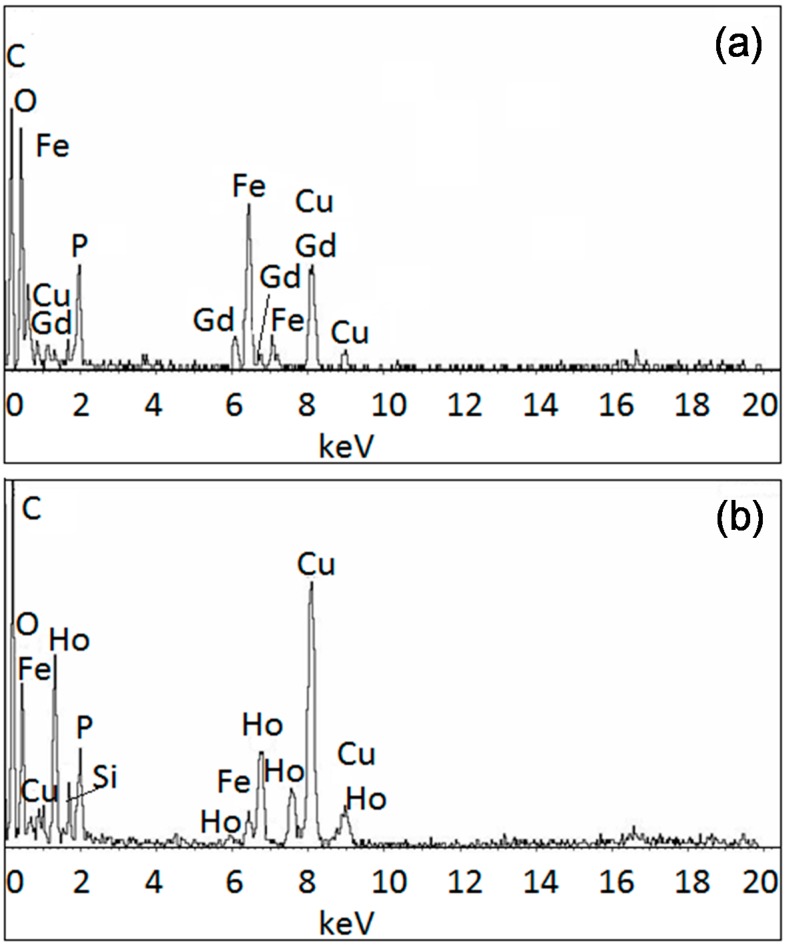
TEM/EDX analysis of the enzymatically-synthesized (**a**) Fe–Gd–O and (**b**) Fe–Ho–O magnetic nanoparticles.

Using SEM/EDX and TEM/EDX, we were able to analyze the composition further for each nanoparticle. As noted above and elsewhere, the compositions of the nanoparticles varied. Figure 4, Figure 5, Figure 6 and Figure 7 provide the analyses of the Fe–Gd–O and Fe–Ho–O magnetic nanoparticles synthesized chemically and enzymatically.

**Figure 8:**
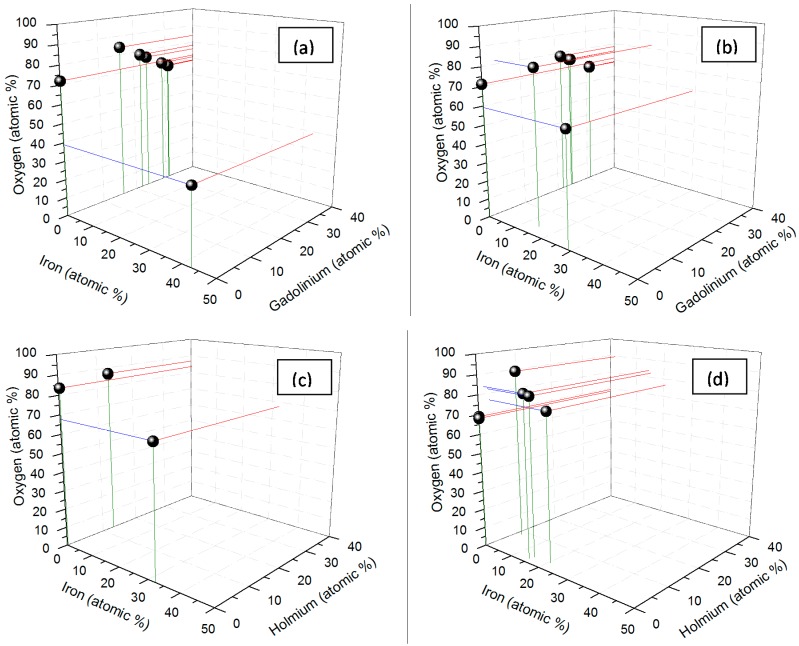
Composition of the nanoparticles determined by TEM-EDX for (**a**) the chemically-synthesized Fe–Gd–O; (**b**) the enzymatically-synthesized Fe–Gd–O; (**c**) the chemically-synthesized Fe–Ho–O; and (**d**) the enzymatically-synthesized Fe–Ho–O. Some points reflect multiple overlapping data.

The deviation of *x*, *y*, and *z* for Fe*_x_*Gd*_y_*O*_z_* and Fe*_x_*Ho*_y_*O*_z_* produced by the two procedures (chemical and enzymatic) might be a reflection of a variation in composition for the individual particles as observed in the TEM/EDX data discussed below. The TEM diffraction data showed that in each sample, some of the nanoparticles were crystalline and some were amorphous (data not shown). The diffraction patterns gathered by the TEM showed a crystalline selected area electron diffraction (SAED) that pointed to the presence of FeO in some, and matched CaO in other nanostructures; the rest of the nanoparticles in each sample revealed amorphous SAED patterns. The varied diffraction patterns observed in each sample indicated that the samples were heterogeneous with respect to composition, which led us to study the composition of these nanoparticles further. To accomplish this task, we isolated about fifteen particles of each of the chemically- and enzymatically-synthesized Fe-Gd-O and the enzymatically-synthesized Fe-Ho-O, and five particles of chemically-synthesized Fe-Ho-O. Figure 8 depicts the clusters of compositions found in the four samples using an *x*, *y*, *z* scatter plot. The plot shows that most of the chemically-synthesized nanoparticles cluster around single-element oxides, and there are only a few nanoparticles that contain all three elements (Fe, Gd, O or Fe, Ho, O). Additionally, none of the chemically-synthesized nanoparticles contained all three components (Fe, Gd, and O or Fe, Ho, and O). However, a small population of the enzymatically-synthesized nanoparticles contained all three elements, suggesting increased synthetic potential of the enzymatic approach.

**Figure 9:**
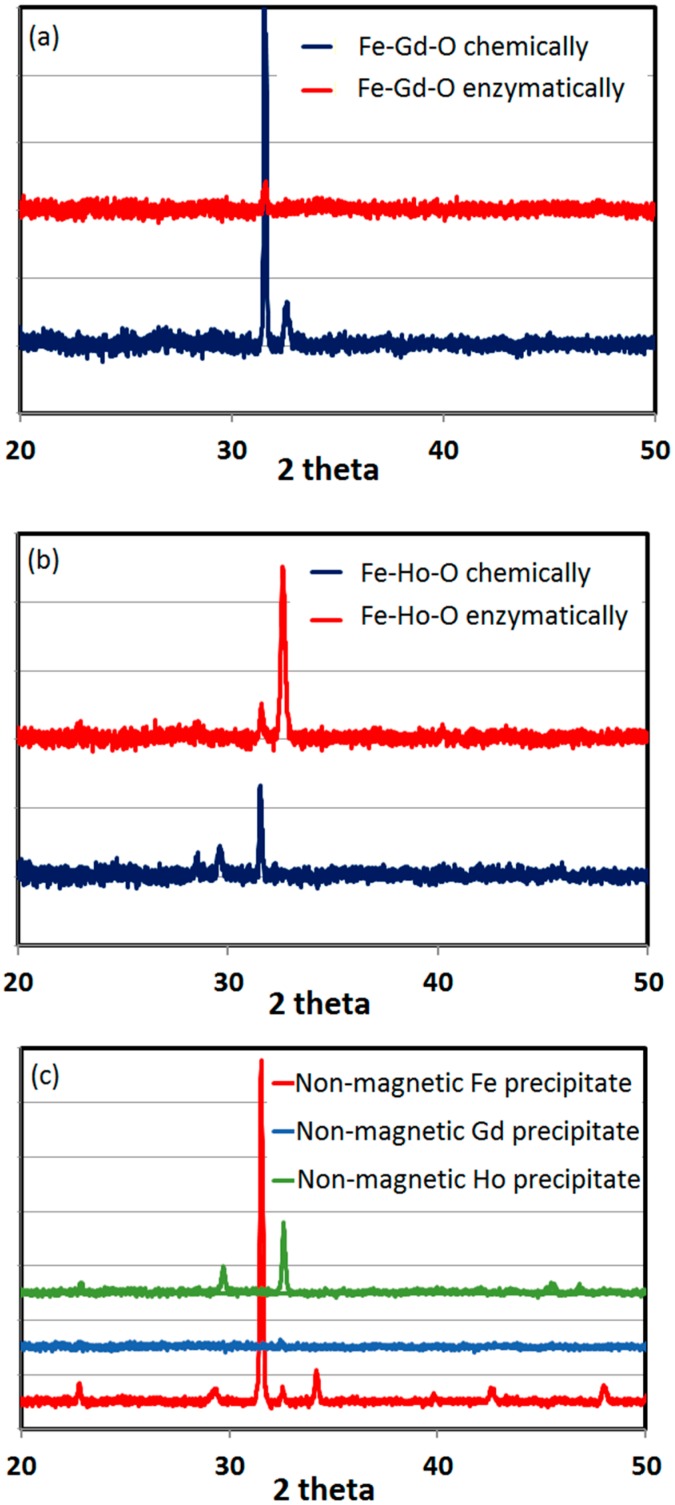
XRD patterns of the (**a**) Fe–Gd–O MNPs; (**b**) Fe–Ho–O MNPs; and (**c**) non-magnetic precipitates obtained by reduction of the chlorides of iron, gadolinium, and holmium.

Figure 9 compares the X-ray diffraction (XRD) patterns of the chemically and enzymatically synthesized Fe–Gd–O and Fe–Ho–O nanoparticles, respectively. These XRD patterns match none of the XRD patterns of the existing Fe–Gd–O and Fe–Ho–O compounds in the Inorganic Crystal Structure Database (ICSD). As noted above, the reduction of the individual iron, gadolinium, and holmium salts using l-ascorbic acid failed to yield magnetic precipitates. However, we characterized the non-magnetic precipitate using XRD and compared it to the magnetic precipitate, as shown in Figure 9. Comparison of the XRD patterns confirms that the magnetic precipitate obtained by using gadolinium and holmium as dopants is distinctly different from the non-magnetic precipitate obtained via reduction of the individual salts.

**Figure 10:**
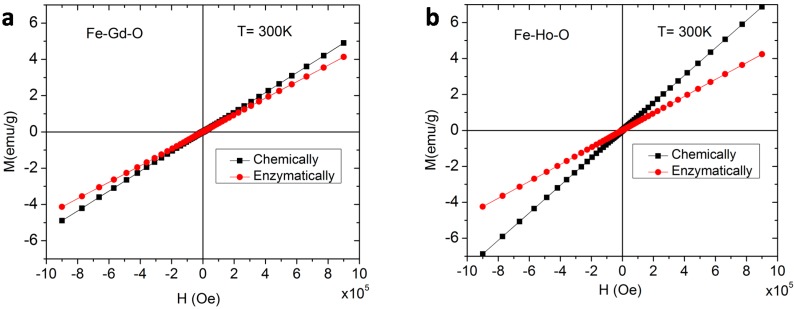
Magnetization curves recorded at 300 K for (**a**) the chemically-synthesized Fe_43±18_Gd_2±0_O_55±18_ MNPs and the enzymatically-synthesized Fe_45±14_Gd_5±2_O_50±15_ MNPs and (**b**) the chemically-synthesized Fe_3±1_Ho_11±2_O_85±3_ MNPs and the enzymatically-synthesized Fe_42±4_Ho_6±4_O_52±5_ MNPs.

**Figure 11:**
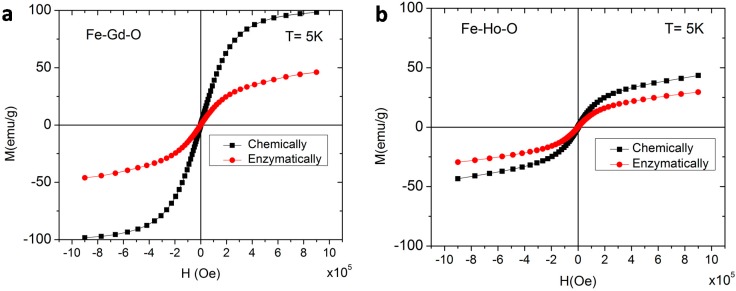
Magnetization curves recorded at 5 K for (**a**) the chemically-synthesized Fe_43±18_Gd_2±0_O_55±18_ MNPs and the enzymatically-synthesized Fe_45±14_Gd_5±2_O_50±15_ MNPs and (**b**) the chemically-synthesized Fe_3±1_Ho_11±2_O_85±3_ MNPs and the enzymatically-synthesized Fe_42±4_Ho_6±4_O_52±5_ MNPs.

The nanoparticles were further characterized by vibrating sample magnetometry (VSM), and the magnetization curves at 300 K for chemically-synthesized and enzymatically-synthesized nanoparticles are shown in Figure 10. In all of these cases, the particles exhibit paramagnetic behavior, since the magnetization increases linearly with increasing magnetic field. At low temperature (5 K), the nanoparticles maintained strong magnetic behavior (Figure 11) and exhibited a significantly higher saturation magnetization of 100 and 45 emu/g for chemically- and enzymatically-synthesized Fe–Gd–O MNPs, and of 50 and 30 emu/g for Fe–Ho–O MNPs. At 300 K, they are paramagnetic; that is, magnetic only under the influence of a magnetic field. At 5 K, each material (regardless of the composition and synthesis method) shows strong magnetic properties with a small coercivity (17–20 Oe) and negligible residual magnetization (0.02 to 0.07 emu/g). The saturation magnetization data are summarized in Table 1 along with coercivity and residual magnetization values.

**Table 1:** Summary of magnetic properties at 5 K.

Composition	Synthesis Method	Saturation Magnetization (emu/g)	Coercivity (Oe)	Residual Magnetization (emu/g)
Fe_43±18_Gd_2±0_O_55±18_	Chemical	100	17	0.07
Fe_45±14_Gd_5±2_O_50±15_	Enzymatic	45	20	0.03
Fe_3±1_Ho_11±2_O_85±3_	Chemical	50	17	0.03
Fe_42±4_Ho_6±4_O_52±5_	Enzymatic	30	17	0.02

**Figure 12:**
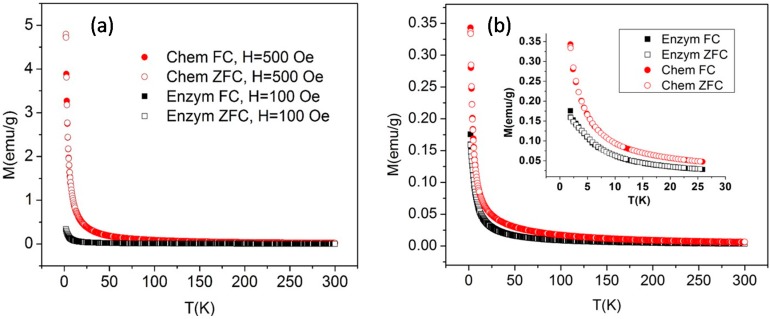
Zero-Field-Cooling (ZFC, open symbols) and Field-Cooling (FC, solid symbols) curves for (**a**) Fe–Gd–O and (**b**) Fe–Ho–O systems.

Figure 12 shows that as the temperature decreases from 300 to 1.9 K, the magnetic behavior of the nanoparticles transforms from paramagnetic to antiferromagnetic, with a Néel temperature around 15–25 K.

On doping with the rare earth elements Gd and Ho, the resulting enzymatically-synthesized nanoparticles were found to be weakly magnetic (~5 emu/g) at 300 K, but with a comparatively higher saturation magnetization of 45 emu/g for Fe_45±14_Gd_5±2_O_50±15_ and 30 emu/g for Fe_42±4_Ho_6±4_O_52±5_ at 5 K. Both chemically and enzymatically synthesized MNPs were observed to be paramagnetic at 300 K and antiferromagnetic under 25 K. Although Gd and Ho possess a higher number of unpaired f electrons as compared to the unpaired d electrons in Fe, enhancement of the magnetic properties by the coupling of these electrons was observed only at low temperature.

The saturation magnetization of the samples might be reduced by the significant presence of non-magnetic precipitates of single-element oxides (as noted earlier, the reduction of individual salts failed to form a magnetic precipitate) with only a small percentage of the MNPs of Fe–Gd–O or Fe–Ho–O present. As previously reported in the case of LnFeO_3_ (Ln = rare earth), phase-selective or homogeneous composition is difficult to achieve during chemical syntheses; the hydrothermal and co-precipitation synthesis of GdFeO_3_ gave an amorphous precipitate, and the combustion route yielded a crystalline powder [30,31]. In another study, the reactant ratios were varied to obtain mono-phasic HoFeO_3_ [32]. Further, a recently reported hydrothermal synthesis optimized the process conditions (alkalinity, reaction temperature, and reaction time) to afford pure phases of GdFeO_3_ and HoFeO_3_, which, however, exhibited weak magnetizations of 0.03 and 0.3 emu/g, respectively [33]. In all of these syntheses, consistent with the chemically- and enzymatically-synthesized nanoparticles described in this paper, the nanoparticles were paramagnetic at room temperature and antiferromagnetic at low temperature. Importantly, the room-temperature magnetization of the nanoparticles described here is significantly greater than that of analogous chemically-synthesized Ln*_x_*Fe*_y_*O*_z_* (Ln = Gd, Ho) samples reported previously [30,31,32].

Our in-house nanoscale GMR sensor can detect one MNP with 60–70 emu/g (sensitivity of 10^−13^ emu); consequently, to detect an analyte using our method, we only need 20 fg MNPs of 5 emu/g. Even with an overall 10% efficiency, this signal would translate to a 10,000-fold improvement in potential limit of detection over conventional ELISA. The synthesized magnetic nanomaterials are not monodisperse. However, polydispersity (population of reporter particles of varied size), is not a substantial barrier to analytical performance. Silver intensification and BCIP/NBT staining are successful examples [14,15,16,17,18,19]. Run-to-run variability, in which a given amount of enzyme bound in the ELISA gives a different magnetic signal, would likely affect analytical performance. We do not see, however, a large variability of this sort in many of the current enzyme-based assays (including all ELISAs and blood glucose monitoring).

## 3. Experimental Section

The chemicals used in the syntheses outlined below were of analytical grade and were used as received from the supplier without further purification. Millipore water (resistivity of >18 MΩ-cm) from a Milli-Q water system was used in the synthesis and washing steps.

### 3.1. Preliminary Experiments of Reduction of Various Metal Salts Using l-Ascorbic Acid

In initial efforts to obtain Fe–Gd–O and Fe–Ho–O precipitates through enzymatic means, we first explored the chemical reduction of ferric chloride, ferric nitrate, cobalt nitrate, nickel sulfate, platinic acid, and copper sulfate. We evaluated the initial matrices and ensured that the precursor concentration was not toxic to the alkaline phosphatase enzyme. In each experiment, 0.1 to 2 mmol of the salt were dissolved in 5 mL Millipore water and evaluated to determine whether a magnetic precipitate was formed upon the addition of l-ascorbic acid. Using this reduction procedure, we obtained precipitates under various experimental conditions of pH (from 6 to 10), temperature (4, 20, 37 °C), and magnetic field conditions during synthesis (presence or absence of a strong bar magnet), but the precipitates from these compounds proved to be non-magnetic. We also observed that although gadolinium and holmium are common components of a permanent magnet, reduction of these rare-earth elements from their chloride salts alone failed to yield magnetic precipitates.

### 3.2. Synthesis of Fe_x_Gd_y_O_z_ and Fe_x_Ho_y_O_z_ Nanoparticles

Ascorbic acid (aa) was used either as purchased (“chemical synthesis” approach) or was produced enzymatically via dephosphorylation of l-ascorbic acid 2-phosphate sesquimagnesium salt hydrate (p-aa) by alkaline phosphatase (AP) (“enzymatic synthesis” approach). Samples of AP were obtained from Sigma (catalog # P6774; 0.049 mL; 3531 units/mg protein and 13 mg protein/mL). One unit activity of AP is defined to hydrolyze 1 μmole of substrate (4-nitrophenyl phosphate) per minute at pH 9.8 at 37 °C. Zeba desalting columns (7 K MWCO from Thermo Fisher Scientific, Rockford, IL, USA) were used to remove more than 95% of the salts (5 mM MgCl_2_ and 0.2 mM ZnCl_2_) present in the AP solution. The enzyme was then resuspended in 100 µL diethanolamine buffer (pH 9.8) containing 5 mM MgNO_3_ and 0.25 mM ZnNO_3_ to give a final concentration of 20 units AP/mL. In a 50-mL centrifuge tube, 0.16 g (0.60 mmol) of FeCl_3_·6H_2_O and 0.05 g (0.1 mmol) GdCl_3_·6H_2_O were dissolved in 5 mL of Millipore water. For chemical or enzymatic synthesis of Fe*_x_*Gd*_y_*O*_z_*, 0.1 g (0.6 mmol) of aa or 0.1 g (0.3 mmol) of p-aa, respectively, were added to the salt solution. In the case of enzymatic synthesis, 15 μL of 60 units/mL AP enzyme were added to the centrifuge tube containing the metal salts. For the synthesis of Fe*_x_*Ho*_y_*O*_z_*, we used a similar procedure with 0.16 g (0.60 mmol) of FeCl_3_·6H_2_O, 0.05 g (0.1 mmol) of HoCl_3_.6H_2_O, 0.1 g (0.6 mmol) of aa (chemical synthesis) or 0.1 g (0.3 mmol) of p-aa (enzymatic synthesis), and 15 μL of 20 units/mL AP enzyme (enzymatic synthesis). The reactions were carried out at 20 °C.

### 3.3. Characterization of Nanoparticles by SEM, TEM, XRD, EDX, and VSM

Nanoparticles were characterized by transmission electron microscopy (TEM; JEOL-2000 FX operating at 200 kV) and equipped with energy dispersive spectrometer (EDX, Oxford Instruments, Abington, UK), scanning electron microscopy (SEM; LEO-1525 operating at 15 kV, Leo (now Carl Zeiss), Oberkochen, Germany), vibrating sample magnetometry (VSM PPMS EverCool II, Quantum Design, Inc., San Diego, CA, USA ), and X-ray diffraction (XRD; D5000 X-ray diffractometer, Siemens (now Bruker), Karlsruhe, Germany). For the TEM analyses, we deposited the nanoparticles suspended in ethanol on a holey carbon film coating a 300-mesh copper grid and allowed them to dry. For the SEM analyses, we deposited them on a silicon wafer and allowed them to dry. We used EDX, XRD, and SAED (selected area electron diffraction, a TEM crystallographic technique) to confirm the composition and phases of the nanoparticles. For the latter studies, a concentrated sample of nanoparticles in ethanol was deposited on a piranha-cleaned glass slide, and XRD was carried out using Cu Kα radiation (λ = 1.540562 Å) in the 2θ range from 0° to 90°.

The magnetic properties (saturation magnetization, residual magnetization, and coercivity) of a known mass of sample were measured using VSM. Saturation magnetization and coercivity were obtained from the hysteresis loop analysis at 300 K and at 5 K. Measurements were recorded with uniform spacing in log field by sweeping the field 100 Oe/s with a maximum applied field up to ±90 kOe. Zero-field-cooling (ZFC) and field-cooling (FC) magnetization curves were measured in the temperature range of 1.9–300 K using a field of 100 Oe. Data were obtained by first cooling the sample from 300 to 1.9 K without applying any magnetic field. To obtain the ZFC curve, a small field of 100 Oe was applied after reaching 1.9 K, and the magnetization was measured at 0.5 K intervals while heating the sample to 300 K with a heating rate of 2 K/min. The FC curve was obtained by cooling the sample from 300 to 1.9 K while keeping the same applied field.

### 3.4. SEM/EDX and TEM/EDX

SEM/EDX and TEM/EDX were used to obtain the composition of the chemically- and enzymatically-synthesized Fe–Gd–O and Fe–Ho–O precipitates. Each SEM/EDX spectrum is an average of at least five samplings, and the average composition with standard deviation was calculated using at least three spectra for each sample. An example of the spectrum obtained for each precipitate is given in the Results and Discussion section.

## 4. Conclusions

In summary, we have demonstrated a novel alternative to optical/electrochemical reporters by enzymatically synthesizing MNPs with higher saturation magnetization than similar nanoparticles (LnFeO_3_ (Ln = Gd, Ho)) synthesized by other routes. This first *in vitro* enzymatic synthesis of magnetic nanoparticles opens a novel approach to magnetic sensing in which the magnetic reporter is enzymatically synthesized *in situ*, thus circumventing any mass-transfer limitations. The enzymatically-synthesized nanoparticles, paramagnetic at 300 K and antiferromagnetic below 25 K, exhibited a strong saturation magnetization, up to 45 emu/g at 5 K. Future optimization of the reaction conditions can potentially lead to a homogeneous composition that reduces (or even eliminates) the presence of precursors or by-product components. Nevertheless, with further optimization of the process parameters, the current precipitated particles can be readily integrated with GMR sensors, such as the one with a reported sensitivity of 10^−13^ emu [2,22,34]. The magnetization of these MNPs and the high sensitivity of the nanoscale GMR [35] offers a 10,000-fold theoretical improvement using this method of magnetic sensing over the conventional optical-based sensing method.

## References

[1] Mani V., Chikkaveeraiah B.V., Rusling J.F. (2011). Magnetic particles in ultrasensitive biomarker protein measurements for cancer detection and monitoring. Expert Opin. Med. Diagn..

[2] Baselt D.R., Lee G.U., Natesan M., Metzger S.W., Sheehan P.E., Colton R.J. (1998). A biosensor based on magnetoresistance technology. Biosens. Bioelectron..

[3] Shieh R., Ackley D.E. (1997). Magnetoresistance-Based Method and Apparatus for Molecular Detection.

[4] Li Y., Srinivasan B., Jing Y., Yao X., Hugger M.A., Wang J.P., Xing C. (2010). Nanomagnetic competition assay for low-abundance protein biomarker quantification in unprocessed human sera. J. Am. Chem. Soc..

[5] Srinivasan B., Li Y., Jing Y., Xu Y.-H., Yao X., Xing C., Wang J.-P. (2009). A detection system based on giant magnetoresistive sensors and high-moment magnetic nanoparticles demonstrates zeptomole sensitivity: Potential for personalized medicine. Angew. Chem. Int. Ed..

[6] Srinivasan B., Li Y., Jing Y., Xing C., Slaton J., Wang J.-P. (2011). A three-layer competition-based giant magnetoresistive assay for direct quantification of endoglin from human urine. Anal. Chem..

[7] Hall D.A., Gaster R.S., Lin T., Osterfeld S.J., Han S., Murmann B., Wang S.X. (2010). GMR biosensor arrays: A system perspective. Biosens. Bioelectron..

[8] Osterfeld S.J., Yu H., Gaster R.S., Caramuta S., Xu L., Han S.J., Hall D.A., Wilson R.J., Sun S., White R.L. (2008). Multiplex protein assays based on real-time magnetic nanotag sensing. Proc. Natl. Acad. Sci. USA.

[9] Hall D.A., Wang S.X., Murmann B., Gaster R.S. Portable biomarker detection with magnetic nanotags. Proceedings of the 2010 IEEE International Symposium on Circuits and Systems (ISCAS).

[10] Gaster R.S., Hall D.A., Nielsen C.H., Osterfeld S.J., Yu H., Mach K.E., Wilson R.J., Murmann B., Liao J.C., Gambhir S.S. (2009). Matrix-insensitive protein assays push the limits of biosensors in medicine. Nat. Med..

[11] Xu L., Yu H., Akhras M.S., Han S.-J., Osterfeld S., White R.L., Pourmand N., Wang S.X. (2008). Giant magnetoresistive biochip for DNA detection and HPV genotyping. Biosens. Bioelectron..

[12] Orlov A.V., Khodakova J.A., Nikitin M.P., Shepelyakovskaya A.O., Brovko F.A., Laman A.G., Grishin E.V., Nikitin P.I. (2013). Magnetic immunoassay for detection of staphylococcal toxins in complex media. Anal. Chem..

[13] Peck R.B., Schweizer J., Weigl B.H., Somoza C., Silver J., Sellors J.W., Lu P.S. (2006). A magnetic immunochromatographic strip test for detection of human papillomavirus 16 E6. Clin. Chem..

[14] Eadie M.J., Tyrer J.H., Kukums J.R., Hooper W.D. (1970). Aspects of tetrazolium salt reduction relevant to quantitative histochemistry. Histochemie.

[15] Altman F.P. (1974). Studies on the reduction of tetrazolium salts—The products of chemical and enzymatic reduction. Histochemie.

[16] Cacao E.E., Nasrullah A., Sherlock T., Kemper S., Kourentzi K., Ruchhoeft P., Stein G.E., Willson R.C. (2013). High-resolution, high-throughput, positive-tone patterning of poly(ethylene glycol) by helium beam exposure through stencil masks. PLoS ONE.

[17] Hainfeld J.F., Liu W. (2008). Site-Specific Enzymatic Deposition of Metal *In Situ* and Use for Chromogenic Immunohistochemical Detection of Diagnostic Biomarkers.

[18] Hainfeld J.F., Liu W. (2008). Binding Oxidoreductases, Peroxidases with Oxidation/Reduction Agents; Immunohistochemistry; Kits. U.S. Patent.

[19] Cacao E.E. (2012). Enzymatic Darkening and Silver Staining: Application in Microfluidic Micro-Retroreflector-Based Heterogeneous Immunoassays. Ph.D. Dissertation.

[20] Wu J., Chumbimuni-Torres K.Y., Galik M., Thammakhet C., Haake D.A., Wang J. (2009). Potentiometric detection of DNA hybridization using enzyme-induced metallization and a silver ion selective electrode. Anal. Chem..

[21] Fanjul-Bolado P., Hernandez-Santos D., Gonzalez-Garcia M.B., Costa-Garcia A. (2007). Alkaline phosphatase-catalyzed silver deposition for electrochemical detection. Anal. Chem..

[22] Kolhatkar A.G., Nekrashevich I., Litvinov D., Willson R.C., Lee T.R. (2013). Cubic silica-coated and amine-functionalized FeCo nanoparticles with high saturation magnetization. Chem. Mater..

[23] (2011). Thermo Scientific Pierce Assay Development Technical Handbook.

[24] Blakemore R. (1975). Magnetotactic bacteria. Science.

[25] Schuler D. (2008). Genetics and cell biology of magnetosome formation in magnetotactic bacteria. FEMS Microbial. Rev..

[26] Liu X., Yun H., Xie J., Huo Z., Wu H., Yang Y. (2013). Research progress of magnetosome formation genes and proteins. Shengwu Jishu Tongbao.

[27] Dohcevic-Mitrovic Z.D., Paunovic N., Radovic M., Popovic Z.V., Matovic B., Cekic B., Ivanovski V. (2010). Valence state dependent room-temperature ferromagnetism in Fe-doped ceria nanocrystals. Appl. Phys. Lett..

[28] Johnson L.M., Thurber A., Anghel J., Sabetian M., Engelhard M.H., Tenne D.A., Hanna C.B., Punnoose A. (2010). Transition metal dopants essential for producing ferromagnetism in metal oxide nanoparticles. Phys. Rev. B Condens. Matter Mater. Phys..

[29] Pool V.L., Klem M.T., Chorney C.L., Arenholz E.A., Idzerda Y.U. (2011). Enhanced magnetism of Fe_3_O_4_ nanoparticles with Ga doping. J. Appl. Phys..

[30] Bedekar V., Jayakumar O.D., Manjanna J., Tyagi A.K. (2008). Synthesis and magnetic studies of nano-crystalline GdFeO_3_. Mater. Lett..

[31] Zhang Y., Zheng A., Yang X., He H., Fan Y., Yao C. (2012). Cubic GdFeO_3_ particle by a simple hydrothermal synthesis route and its photoluminescence and magnetic properties. CrystEngComm.

[32] Jiang L., Liu W., Wu A., Xu J., Liu Q., Qian G., Zhang H. (2012). Low-temperature combustion synthesis of nanocrystalline HoFeO_3_ powders via a sol-gel method using glycin. Ceram. Int..

[33] Zhou Z., Guo L., Yang H., Liu Q., Ye F. (2014). Hydrothermal synthesis and magnetic properties of multiferroic rare-earth orthoferrites. J. Alloy. Compd..

[34] Wirix-Speetjens R., Reekmans G., de Palma R., Liu C., Laureyn W., Borghs G. (2007). Magnetoresistive biosensors based on active guiding of magnetic particles towards the sensing zone. Sens. Actuators B.

[35] Litvinov D., Willson R. (2013). Nanomagnetic Detector Array for Biomolecular Recognition. U.S. Patent.

